# Transthoracic echocardiography for the diagnosis of left ventricular thrombosis in the postoperative care unit

**DOI:** 10.1186/cc10025

**Published:** 2011-02-09

**Authors:** Theodosios Saranteas, Anastasia Alevizou, Maria Tzoufi, Fotios Panou, Georgia Kostopanagiotou

**Affiliations:** 1 Department of Anaesthesia and Cardiovascular Critical Care, Medical School, University of Athens, Attikon Hospital of Athens, Haidari, Rimini Str 1, 12462, Haidari, Athens, Greece; 2 Department of Cardiology, Medical School, University of Athens, Attikon Hospital of Athens, Haidari, Rimini Str 1, 12462, Haidari, Athens, Greece

## Abstract

**Introduction:**

Transthoracic echocardiography (TTE) is a reliable, noninvasive imaging method that is useful in the evaluation of cardiovascular thrombosis. We conducted a retrospective study of all the echocardiograms from patients in the postoperative care unit to assess the role of TTE in thrombus identification in the left ventricle.

**Methods:**

This retrospective database evaluation included all echocardiograms during a 14-month period. The echocardiographic examination protocol included the subcostal four-chamber view, the apical four-chamber view, the apical two-chamber view and the parasternal view, along the long and short axes in both spontaneously and mechanically ventilated patients. All echocardiograms were obtained within the 48 hours immediately following surgery.

**Results:**

In total, 160 postoperative echocardiograms were obtained from 160 patients and resulted in the detection of five cases of left ventricular thrombosis. Subgroup analysis showed that 21 and 35 of the 160 patients examined had either dilated or ischemic cardiomyopathy, respectively. In these patients, preoperative echocardiograms had been obtained recently prior to surgery and were negative for left ventricular thrombus. In three of 35 patients with ischemic cardiomyopathy and two of 21 patients with dilated cardiomyopathy, thrombus was identified in the left ventricle. The thrombi were mobile, uncalcified and pedunculated and were located in the apex of the left ventricle. In addition, no clinical consequences of the left ventricular thrombi were recorded.

**Conclusions:**

Low-flow conditions in heart chambers due to ischemic or dilated cardiomyopathy in conjunction with the hypercoagulability caused by perioperative prothrombotic factors may lead to thrombotic events in the left ventricle.

## Introduction

Both transesophageal echocardiography (TEE) and transthoracic echocardiography (TTE) can identify the cause of shock and other lesions in the setting of the intensive care unit (ICU). Echocardiography can significantly alter the management of up to 46% of critically ill patients [1-4]. TTE also offers a noninvasive way to evaluate cardiac function. Traditionally, this role has been performed solely by cardiologists with extensive training in advanced TTE techniques. However, a growing body of evidence points to the ability of noncardiologist intensivists to employ TTE in the ICU setting [5-8]. Anesthesiologists with a cardiac and echocardiography background can successfully perform TTE in almost all patients when necessary, and they typically provide valuable diagnostic information of critical importance [9-11]. Recently, Jensen *et al*. [9] have advocated the position that TTE is the only technique that provides dynamic real-time bedside imaging of the heart. At least one usable window for cardiac imaging can be obtained in 97% of a mixed ICU population, and TTE results contribute conclusive information in 25% of these cases [9].

In addition, focused ultrasonography has emerged as an important and noninvasive bedside diagnostic tool for the emergency room physician that facilitates the early detection of potentially reversible and time-dependent conditions. Currently, the two primary indications for TTE are the diagnosis of pericardial tamponade and the confirmation or refutation of pulseless electrical activity [8].

In this retrospective study, we report our experience using TTE for thrombus identification in the left ventricle in the setting of a postoperative care unit.

## Materials and methods

We conducted a retrospective study of all echocardiograms from patients in the postoperative care unit to assess the role of TTE in thrombus identification in the left ventricle during the postoperative period. The postoperative care unit receives both elective and emergency admissions from a wide range of surgical specialties, including major vascular, thoracic, abdominal and orthopedic surgery.

Because of the retrospective design of the study, formal research ethics committee approval and patients' written informed consent for publication of this manuscript and accompanying images were deemed unnecessary after consultation with the local ethics committee.

A retrospective database evaluation was undertaken for all echocardiograms obtained during a selected 14-month period. All echocardiograms examined were part of a specific echocardiographic protocol that is performed as a local standard of care for typical postoperative indications, including evaluation of left and right ventricular function, hypotension, pulmonary edema, diagnosis of pericardial effusion, suspected valvular disease and refractory hypoxia.

A competent anesthetist consultant who was undertaking European Society of Echocardiography accreditation performed the TTE studies. Hand-carried ultrasound examinations were conducted using a 2- to 5-MHz phased array transducer on a portable ultrasound unit (Vivid I; GE Healthcare, Waukesha, Wisconsin, USA).

The echocardiographic examination protocol included visualization of the subcostal four-chamber view, the apical four-chamber view, the apical two-chamber view and the parasternal long-axis and short-axis views in either spontaneously breathing or mechanically ventilated patients.

All results were digitally archived to permit peer review, and consultant cardiologists reviewed ambiguous results.

## Results

All echocardiography was performed within the 48 hours immediately following surgery. During the 14-month period, 160 postoperative echocardiograms were obtained from 160 patients (85 females and 75 males) who had a median age of 67 years and who ranged in age from 20 to 89 years. In total, 125 of the 160 patients were spontaneously breathing, whereas the remaining 35 patients were under mechanical ventilation. The echocardiograms were obtained from patients who had undergone the following operations: major thoracic (10 patients), orthopedic (40 patients), vascular (35 patients) or abdominal (75 patients). In 45 of the 75 abdominal operations, the incision was in or extended into the upper abdomen.

Five of 160 patients were found to have masses consistent with thrombi in the left ventricle. All of the thrombi were detected in the acute setting when TTE was performed and, specifically, were identified in the left ventricular apex. Table 1 shows the characteristics and findings for these five patients.

**Table 1:** Patient characteristics^a^

Patients	Type of ventilation	Type of surgery	Cardiac pathology	Thrombus location
1	SB	Vascular surgery (axillary-femoral artery bypass)	Dilated cardiomyopathy EF = 25%	Left ventricle
2	SB	Vascular surgery (aneurysm of the abdominal aorta)	Dilated cardiomyopathy EF = 25%	Left ventricle
3	MV	Abdominal surgery (ischemic colitis-bowel resection)	Anterior myocardial infarction, ischemic cardiomyopathy: EF = 35%	Left ventricle
4	MV	Abdominal surgery (cancer, bowel perforation)	Anterior myocardial infarction, ischemic cardiomyopathy EF = 30%	Left ventricle
5	MV	Abdominal surgery (cancer, bowel perforation)	Anterior-posterior myocardial infarction, ischemic cardiomyopathy EF = 30%	Left ventricle

^a^SB, spontaneous breathing; MV, mechanical ventilation; EF, ejection fraction.

**Figure 1:**
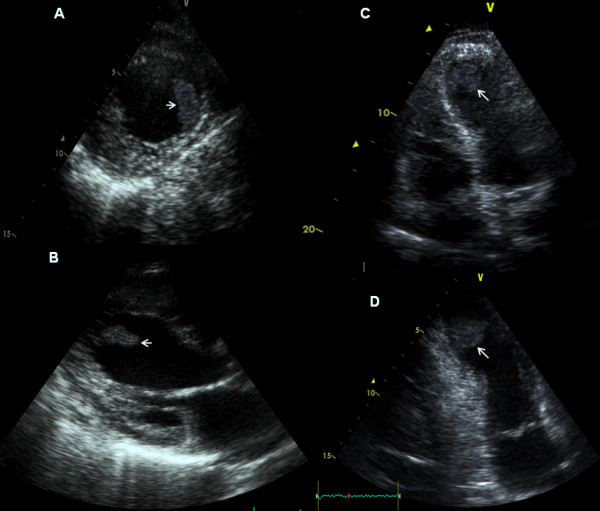
**Transthoracic echocardiography of the left ventricle**. Thrombus (arrows) in the apex of the left ventricle is clearly depicted in the **(A) **short, **(B) **long parasternal axes as well as in the **(C) **apical four-chamber and in the **(D) **apical two-chamber views.

Clear visualization of the thrombi was observed in the apical four-chamber view, the apical two-chamber view and the parasternal short-axis view. In one of the cases, the thrombus was also visualized in the parasternal long-axis view (Figure 1). Further examination revealed that the thrombi were mobile, uncalcified, pedunculated and protruding into the left ventricle.

**Figure 2:**
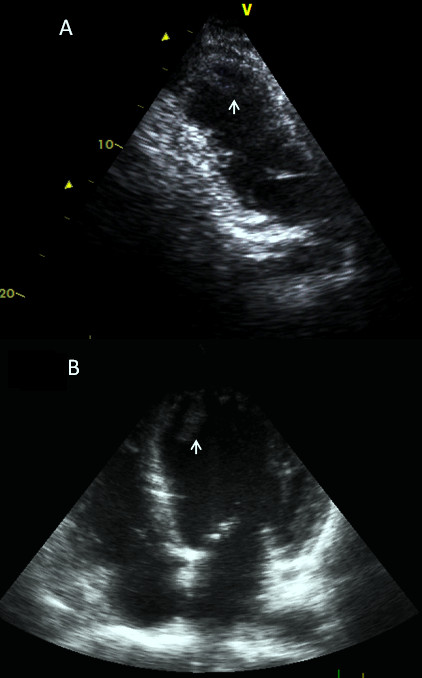
**Transthoracic echocardiography of the left ventricle**. Thrombus (arrows) in the apex of the left ventricle in **(A) **patients with ischemic cardiomyopathy and **(B) **patients with dilated cardiomyopathy.

Subgroup analysis showed that 21 and 35 of 160 patients examined had dilated and ischemic cardiomyopathy, respectively. In these patients, preoperative echocardiograms had been obtained recently prior to surgery and were negative for left ventricular thrombus. In 3 (8.5%) of 35 patients with ischemic cardiomyopathy and 2 (9.52%) of 21 patients with dilated cardiomyopathy, thrombus was identified in the left ventricle (Figure 2). In addition, we did not observe any clinical consequences related with the left ventricular thrombi, that is, thromboembolic events.

After diagnosis of thrombus in the left ventricle, full anticoagulant treatment with low-molecular-weight heparin was started.

## Discussion

Cardiovascular thrombosis is common in the ICU setting because critically ill patients are immobile and sedated, exposed to thrombin-generating procedures (for example, central venous catheterization) and frequently have other thrombotic risk factors (for example, malignancies or trauma) [12,13].

Patients at risk for the development of a left ventricular thrombus are readily identified with echocardiography. Thrombi generally involve the apex of the left ventricle, most often in the presence of akinesis or dyskinesis. Although myocardial infarction is the most common predisposing cause of left ventricular thrombi, left ventricular thrombi can develop in any situation in which low flow occurs [14].

In our study, all of the left ventricular thrombi were detected in the acute setting when TTE was performed immediately after surgery for hemodynamic monitoring purposes. In the left ventricle, thrombi were located only at the cardiac apex. Ischemic cardiomyopathy in three patients and dilated cardiomyopathy in two patients may have contributed to this thrombus formation [14]. Although all of our patients showed increased procoagulant activity, it was generally difficult to explain the origin of the cardiovascular thrombi. The nature of the thrombi (soft, mobile and uncalcified, forming a mass along the akinetic and/or dyskinetic cardiac wall), along with the negative left ventricular thrombus findings on preoperative echocardiograms, led to the conclusion that the thrombi were formed during the perioperative period and that perioperative prothrombotic factors together with the patients' prothrombotic substrates contributed to the thrombotic events. More specifically, in our patients, low-flow conditions due to ischemic or dilated cardiomyopathy [14] in conjunction with the hypercoagulability caused by surgical trauma [15] and/or cancer [16] might have contributed to left ventricular thrombosis.

Although surgical patients may have absolute contraindications for anticoagulant therapy immediately after surgery, it is not well known whether the nature, quality and presence of thrombus represent an absolute indication for full-dose anticoagulant treatment. In our cases, TTE revealed an uncalcified, fresh and extensively mobile structure in the apex of the left ventricle. Therefore, full-dose anticoagulant therapy was considered indispensable in avoiding the consequences of thrombus dislodgement into the bloodstream.

The sensitivity of TTE for detecting left ventricular thrombosis ranges between 92% and 95%, with specificity of 86% to 88% [17]. On the contrary, in TEE, midesophageal apical planes did not place the left ventricular apex in the near field, which is optimal for this purpose; transgastric views cannot always be obtained, especially in awake patients, and are often of low quality [18,19].

In TTE, large, protruding and highly mobile thrombi are readily seen from the apical window, while laminar thrombi that do not protrude into the cavity are likely to be missed [20]. Poor imaging quality also reduces the accuracy of thrombus identification and may produce both false-negative and false-positive results. An additional method of confirming the presence or absence of left ventricular thrombosis, especially in cases of poor imaging quality, is to use contrast enhancement for left ventricular opacification [21]. In our five patients, there was excellent delineation of the left ventricular cavity and clear depiction of the ventricular apical thrombus. In addition, vague results were thoroughly examined by consultant cardiologists, competent in TTE, who deemed the use of ultrasound contrast agents unnecessary.

In our study, thrombi in the left ventricle were mainly identified in the apical four-chamber view and by scanning the apex in the short-axis parasternal view. Using the long-axis parasternal view, only one case of left ventricle thrombosis was recognized. From this view, it was not always possible to visualize the left ventricular apex. Indeed, only when the transducer is moved to a lower interspace is the left ventricular apex included in the field [22]. Among our patients, a view of the left ventricular apex was obtained only with difficulty by using the long-axis parasternal view because of the fact that our patients were always supine and could not be moved into the left lateral decubitus position.

In addition, left ventricular thrombi could not be seen using the subcostal view. In the first, second and third patients, the main reasons were technical limitations related to foreshortening and to the inability to visualize the left ventricular apex from the subcostal view because of the position of the transducer relative to the cardiac apex [22,23]. In the fourth and fifth patients, extension of the surgical incision into the upper abdomen made it impossible to record this view.

## Study limitations

Although this study is retrospectively designed, our observations offer important information in an otherwise unknown topic such as that of left ventricular thrombosis in surgical patients; hence, the hypothesis of the high rates of left ventricular thrombi in surgical patients with either ischemic or dilated cardiomyopathy remains to be confirmed in a prospective study.

## Conclusions

There is clear value in using TTE imaging of the heart for the purpose of cardiovascular evaluation and optimization in the postoperative period. This report demonstrates that information gained from TTE imaging contributes to the correct diagnosis of cardiovascular thrombosis in patients in the acute postoperative setting who have either dilated or ischemic cardiomyopathy.

## Key messages

• TTE can provide serendipitous information in critical care patients and could also facilitate the diagnosis of systemic or local disorders.

• Low-flow conditions in the heart chambers due to ischemic or dilated cardiomyopathy in conjunction with the hypercoagulability caused by surgical trauma and/or cancer may lead to left ventricular thrombosis during the perioperative period. The thrombi formed were located in the left ventricular apex and were mainly depicted in the apical four- and two-chamber views as well as in the parasternal short-axis view.

## Abbreviations

ICU: intensive care unit; TEE: transesophageal echocardiography; TTE: transthoracic echocardiography.

## Competing interests

The authors declare that they have no competing interests.

## Authors' contributions

TS conceived of the study and performed all the echocardiography. AA provided analysis of ultrasound imaging data and drafted the manuscript. MT reviewed and archived ultrasound data. FP provided expert echocardiographic consulting. GK participated in the design of the study.
